# The Significance of the Sulfatase Pathway for Local Estrogen Formation in Endometrial Cancer

**DOI:** 10.3389/fphar.2017.00368

**Published:** 2017-06-23

**Authors:** Maša Sinreih, Tamara Knific, Maja Anko, Neli Hevir, Katja Vouk, Aleš Jerin, Snježana Frković Grazio, Tea Lanišnik Rižner

**Affiliations:** ^1^Institute of Biochemistry, Faculty of Medicine, University of LjubljanaLjubljana, Slovenia; ^2^Institute of Clinical Chemistry and Biochemistry, University Medical CentreLjubljana, Slovenia; ^3^Division of Obstetrics and Gynecology, Department of Pathology, University Medical CentreLjubljana, Slovenia

**Keywords:** aromatase, 17β-hydroxysteroid dehydrogenases, aldo-keto reductase 1C3, sulfotransferases, sulfatase

## Abstract

Endometrial cancer (EC) is the most common estrogen-dependent gynecological malignancy in the developed World. To investigate the local formation of estradiol (E2), we first measured the concentrations of the steroid precursor androstenedione (A-dione) and the most potent estrogen, E2, and we evaluated the metabolism of A-dione, estrone-sulfate (E1-S), and estrone (E1) in cancerous and adjacent control endometrium. Furthermore, we studied expression of the key genes for estradiol formation via the aromatase and sulfatase pathways. A-dione and E2 were detected in cancerous and adjacent control endometrium. In cancerous endometrium, A-dione was metabolized to testosterone, and no E2 was formed. Both, E1-S and E1 were metabolized to E2, with increased levels of E2 seen in cancerous tissue. There was no significant difference in expression of the key genes of the aromatase (*CYP19A1*) and the sulfatase (*STS, HSD17B1, HSD17B2*) pathways in cancerous endometrium compared to adjacent control tissue. The mRNA levels of *CYP19A1* and *HSD17B1* were low, and *HSD17B14*, which promotes inactivation of E2, was significantly down-regulated in cancerous endometrium, especially in patients with lymphovascular invasion. At the protein level, there were no differences in the levels of STS and HSD17B2 between cancerous and adjacent control tissue by Western blotting, and immunohistochemistry revealed intense staining for STS and HSD17B2, and weak staining for SULT1E1 and HSD17B1 in cancerous tissue. Our data demonstrate that in cancerous endometrium, E2 is formed from E1-S *via* the sulfatase pathway, and not from A-dione *via* the aromatase pathway.

## Introduction

Endometrial cancer (EC) is the fifth-most-common cancer in women in Western Europe and the USA, with the majority of cases arising after menopause (Colombo et al., 2016; Morice et al., 2016). EC can be classified into estrogen-dependent type I, which comprises 80% of all cases, and the poorly differentiated, more aggressive, type II. Although, type II EC was considered to be estrogen independent (Inoue, 2001; Samarnthai et al., 2010), experimental data suggest involvement of estrogens (Berstein et al., 2003; Wan et al., 2016).

**Figure 1:**
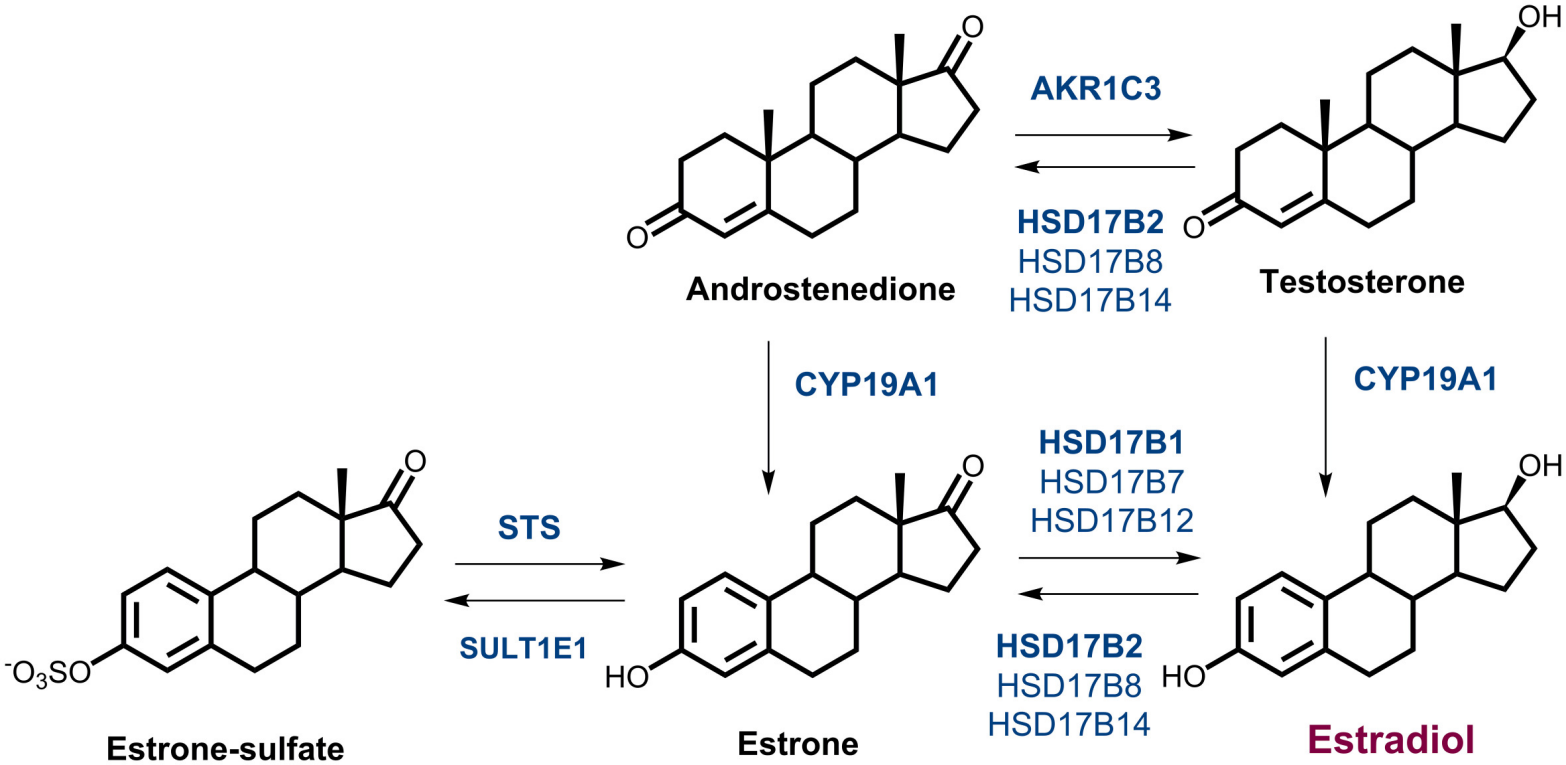
Estrogen biosynthesis. Formation of estrogens via the aromatase pathway from androstenedione and testosterone, by the actions of aromatase (CYP19A1), and the reductive 17β-hydroxysteroid dehydrogenases types 1, 7, and 12 (HSD17B1, HSD17B7, HSD17B12) and type 5 (AKR1C3). Formation of estrogens via the sulfatase pathway from estrone-sulfate, by the action of sulfatase (STS), and the reductive HSD17B1, HSD17B7, and HSD17B12. The oxidative 17β-HSD types 2, 4, 8, and 14 (HSD17B2, HSD17B4, HSD17B8, HSD17B14) catalyze the inactivation of estradiol to estrone and sulfotransferase (SULT1E1) catalyzes conjugation of estrone.

Local estrogen formation has an important role in the development of EC and increased estradiol (E2) concentrations have been detected in cancerous, as compared to normal endometrium (Berstein et al., 2003). Locally, E2 can be formed either via the so-called aromatase pathway from androstenedione (A-dione), which originates from dehydroepiandrosterone-sulfate (DHEA-S) and DHEA, or from testosterone (T), by the actions of aromatase and the reductive 17β-hydroxysteroid dehydrogenases (enzymes 17β-HSD, HSD17B; Figure 1). These are NADPH dependent enzymes, which due to high intracellular concentration ratio NADPH/NADP^+^ act preferentially as reductases in a cellular context (Agarwal and Auchus, 2005). The most potent estrogen, E2 can also be formed from estrone-sulfate (E1-S) via the sulfatase pathway by the actions of sulfatase (STS) and the reductive enzymes HSD17B (Figure 1).

The aromatase pathway depends on availability of A-dione or T. A-dione, with 1–8 nM concentrations in blood, originates mainly from adrenal gland (zona reticularis), from ovaries in premenopausal women and also from conversions of DHEA-S and DHEA in peripheral tissues. Aromatase (CYP19A1) converts A-dione and T into estrone (E1) and E2, respectively (Krekels et al., 1991). As the plasma concentrations of A-dione in postmenopausal women are ~4-fold higher than those of T (Simpson, 2002; Keefe et al., 2014), aromatase mainly converts A-dione to E1. Currently, the data on aromatase expression in EC are controversial, with everything from high levels, to no significant differences between diseased and normal tissues, to no expression being reported (Yamamoto et al., 1993; Watanabe et al., 1995; Berstein et al., 2005; Jongen et al., 2005; Segawa et al., 2005; Lanišnik Rižner et al., 2006; Pathirage et al., 2006; Smuc and Rizner, 2009; Takahashi-Shiga et al., 2009; Lepine et al., 2010; Cornel et al., 2012).

E1 formed from A-dione should be further activated by the reductive estrogenic 17β-HSD type 1 (HSD17B1), to form E2. Although, several groups have failed to detect expression of *HSD17B1* in normal and cancerous endometrium, others have seen low mRNA levels in both tissues (Casey et al., 1994; Zeitoun et al., 1998; Utsunomiya et al., 2001; Lanišnik Rižner et al., 2006; Smuc and Rizner, 2009) with decreased mRNA levels in EC compared to adjacent control endometrial tissue (Smuc and Rizner, 2009; Lepine et al., 2010). In contrast Cornel et al. (2012) showed increased mRNA levels of *HSD17B1* in ERα positive grade 1 EC compared to control tissue.

In addition to HSD17B1, three other reductive estrogenic 17β-HSDs, types 7 and 12 (HSD17B7 and HSD17B12), and type 5 (aldo-keto reductase 1C3, AKR1C3), that can form E2 from E1, albeit with lower catalytic efficiencies, can contribute to E2 formation. Also the expression of these genes in EC is rather controversial as has been reviewed (Rižner, 2013). There were no significant differences in mRNA levels of *AKR1C3* (Rizner et al., 2006; Smuc and Rizner, 2009; Cornel et al., 2012), the expression of *HSD17B7* was reported as decreased (Smuc and Rizner, 2009) or unchanged (Lepine et al., 2010; Cornel et al., 2012) and expression of *HSD17B12* was unchanged (Smuc and Rizner, 2009; Cornel et al., 2012) or increased (Lepine et al., 2010) in EC compared to control tissue.

The expression of the oxidative NAD^+^ dependent estrogenic 17β-HSDs, types 2, 4, 8, and 14 (HSD17B2, HSD17B4, HSD17B8, and HSD17B14), can also affect local E2 concentrations. These enzymes catalyze inactivation of E2 to E1. Previous studies by Lepine et al., and our group revealed increased mRNA levels of *HSD17B2* (Lepine et al., 2010; Sinreih et al., 2013) in EC, while Cornel et al. found no significant difference in ERα positive grade 1 EC (Cornel et al., 2012). For *HSD17B4* and *HSD17B8* we saw no changes in gene expression in EC compared to adjacent control tissue (Smuc and Rizner, 2009), while expression of *HSD17B14* has not yet been studied in EC.

E2 can also be formed *via* the sulfatase pathway from E1-S by the actions of STS and the reductive enzymes HSD17B1, HSD17B7, HSD17B12, and also AKR1C3. Unchanged (Smuc and Rizner, 2009), and increased (Lepine et al., 2010) expression of *STS* have previously been reported in EC. Sulfotransferase SULT1E1 catalyzes conjugation of estrogens and our previous studies show that gene encoding this enzyme is not differentially expressed in EC as compared to adjacent control tissue (Hevir et al., 2011b), while Lepine et al., reported borderline increased mRNA levels in cancer tissue (Lepine et al., 2010).

There is a great need for a better understanding of the local formation of E2 in cancerous endometrium, which may reveal novel targets for treatment of this most common gynecological malignancy. The aims of the present study were thus to investigate E2 formation in paired samples of EC and adjacent control endometrium at different levels. Our goals were: (i) to determine concentrations of steroid precursor A-dione and the most potent estrogen E2, (ii) to examine capacity for formation of A-dione, E1-S and E1; (iii) to re-examine the mRNA levels of individual genes involved in the aromatase pathway and the sulfatase pathway of E2 formation, (iv) to evaluate protein levels of the key players in the sulfatase pathway, STS, SULT1E1, HSD17B2, and HSD17B1 and their prognostic potential.

## Materials and methods

### Endometrial tissue

The specimens of EC and paired adjacent control endometrium were obtained from 55 patients undergoing hysterectomies for histologically proven EC (Table 1, Supplementary Table 1). The study was approved by the National Medical Ethics Committee of the Republic of Slovenia with written informed consent required from all subjects involved. The patients were all treated in the Department of Gynecology and Obstetrics at the University Medical Centre Ljubljana, from 2003 to 2010. The samples used for steroid concentration measurements, metabolism studies, qPCR, Western blotting and immunohistochemical staining have been selected chronologically.

**Table 1:** Demographic and histopathological characteristics of the endometrial cancer patients.

**Characteristics**	**Patient sample size**	**Mean ± *SD***
Age (years)	55	62.0 ± 13.7
Weight (kg)	53	82.2 ± 18.2
Height (cm)	49	163.1 ± 5.2
BMI (kg/m^2^)	49	30.9 ± 7.0
**MENOPAUSAL STATUS**		
Premenopausal	16	
Postmenopausal	38	
NA	1	
Endometrioid adenocarcinoma	45	
Papillary serous carcinoma	6	
Carcinosarcoma	1	
Dedifferentiated	3	
**GRADE**		
G1	32	
G2	8	
G3	5	
**INVASION OF MYOMETRIUM**		
<50%	39	
≥50%	14	
NA	1	
**PRESENCE OF LYMPHOVASCULAR INVASION**		
No	39	
Yes	13	
NA	2	
**FIGO STAGE**		
IA	37	
IB	11	
II	1	
III	3	
IV	2	
NA	1	

*G1–G3 endometrioid adenocarcinoma; NA, data not available*.

### Steroid concentration measurements

Ten paired samples of EC and adjacent control tissue were frozen in liquid nitrogen and ground to a fine powder. Prior to extraction, 100–200 mg of homogenate was suspended in 0.1 M sodium phosphate buffer (pH 7.4). The extraction was performed three times with 4 mL of diethyl ether; the extracts were pooled and evaporated under a stream of nitrogen. Before analysis, the samples were resuspended in phosphate buffer. The levels of A-dione were measured using a double antibody radioimmunoassay with interassay CV < 10%, which uses a [^125^I]-labeled tracer (Diagnostic Systems Laboratories, Webster, TX, USA). E2 was determined using an automated chemiluminescent immunoassay (Liaison, Diasorin, Saluggia, Italy) with interassay CV < 14%. Concentrations were calculated as pmol/g powdered tissue. The limit of detection was 0.1 pmol/g for A-dione and 0.4 pmol/g for E2.

### RNA isolation and qPCR

Total RNA was isolated from tissue samples using the Tri Reagent (Sigma Aldrich, St. Louis, MI, USA), according to the manufacturer instructions. The quality of the RNA samples was determined using an Agilent 2100 Bioanalyzer where they showed an average RIN of 7.7. The total RNA was reverse transcribed using SuperScript® VILO™ cDNA Synthesis kit (Invitrogen, Thermo Fisher Scientific, Carlsbad, CA, USA). One microgram of total RNA was converted into cDNA (20 μL) according to the manufacturer instructions, and then stored at −20°C. *CYP19A1, STS, HSD17B1*, and *HSD17B14* mRNA expression levels were determined with the exon-spanning hydrolysis probes (FAM or VIC dye labeled) that are commercially available as “Assay on Demand” (Applied Biosystems, Foster City, CA, USA). The qPCR analysis was performed in 27 samples of EC and adjacent control endometrium for *STS* and *HSD17B1*, in 22 paired samples for *CYP19A1* and 21 paired samples for *HSD17B14. PPIA, HPRT1*, and *POLR2A* were selected as the most stable reference genes, as described previously (Hevir et al., 2011a). The assay details are shown in Supplementary Table 2. The gene expression for each sample was calculated from the crossing point value (Cp) as E^−Cp^, divided by the normalization factor and multiplied by 10^14^. For *HSD17B14* and *CYP19A1*, only *PPIA* was used as the reference gene for the relative quantification with the comparative Ct method. Expression levels were multiplied by 10^14^.

### Western blotting

Proteins were isolated from samples of EC tissue and the adjacent control endometrial tissue previously used for RNA isolation, following the Tri Reagent instructions. Protein concentrations were determined by Bradford assay and protein aliquots of 30 μg were separated by SDS PAGE on 10% Tris-glycine gels. The proteins were transferred from gels to polyvinylidene difluoride membranes (Millipore Corporation, Billerica, MA, USA) and incubated with 5% non-fat milk in Tris Buffered Saline buffer, with 0.1% Tween® 20 (TTBS) or with 5% BSA in TTBS, both for 2 h, when evaluating STS. The membranes were then incubated with antibodies against STS, HSD17B2, SULT1E1, and HSD17B1 and GAPDH, as normalization control, using antibodies and protocols described in Supplementary Table 3 (Dibbelt and Kuss, 1986; Dibbelt et al., 1989). Protein extracts from model cell lines of control endometrium (HIEEC), EC (HEC-1A), and liver cancer (HepG2) were used as controls.

Supersignal™ West Pico Chemiluminiscence Substrate (Thermo Fischer Scientific, Life Tecnologies, Carlsbad, CA, USA) was used for detection of the bound antibodies, according to the manufacturer instructions, using a Fujifilm LAS4000 image reader (Fujifilm, Tokyo, Japan). Detection of GAPDH was used as the normalization control. Quantification of the Western blotting was carried out with ImageJ (National Institutes of Health, USA) or the Multi Gauge software (Fujifilm software, Fujifilm, Tokyo, Japan).

### Steroid hormone metabolism studies

#### A-dione metabolism

From 60 to 85 mg of homogenized tissue from seven paired samples was resuspended in 470 μL of 50 mM sodium phosphate buffer pH 7.4 with protease inhibitor cocktail (Sigma Aldrich, St. Louis, MI, USA) added. This mixture was incubated with 80 nM androst-4-ene-3,[1,2,6,7-^3^H(N)]-,17-dione in the presence of 2 mM NADPH in a reaction volume of 500 μL, for 20 h at 37°C. The steroids were extracted with ethyl acetate (3 × 500 μL), dried, resuspended in 40 μL ethyl acetate, and applied to Whatman Partisil® LK6DF silica gel TLC plates. The chromatograms were developed in cyclohexane/ethyl acetate (1:1, v/v), followed by autoradiography using Kodak BioMax MS-Films and LE Intensifying Screen (Sigma Aldrich, St. Louis, MI, USA) with incubation at −80°C for 6 days. The bands were identified by co-migration with authentic standards. Samples were next re-extracted from silica gel with ethyl acetate (3 × 500 μL), dried, resuspended in 40 μL ethyl acetate, applied to new silica gel TLC plates, and developed in cyclohexane/ethanol (95:5, v/v).

Additionally, 24 mg of homogenized tissue from three paired samples were incubated with 8 nM androst-4-ene-3,[1,2,6,7-^3^H(N)], 17-dione in 50 mM sodium phosphate buffer, pH 7.4, with protease inhibitor cocktail (Sigma Aldrich, St. Louis, MI, USA) and 2.6 mM NADP^+^, 5 mM Glucose-6-Phosphate, and 2.5 U of GAPDH. After 20 h incubation at 37°C the steroids were extracted from the medium with ethyl acetate (3 × 500 μL), dried in a SpeedVac™, and resuspended in 50% acetonitrile in water. The samples were then analyzed by HPLC using a Kinetex 2.6 μ XB-C18 column (150 × 4.6 mm; Phenomenex; Aschaffenburg, Germany) equipped with a Securityguard guard column and Securityguard cartridges (C18; 4 × 3.0 mm; Phenomenex, Aschaffenburg, Germany). The mobile phase was acetonitrile: water, 1:1, and the flow rate was 0.7 mL/min. The column temperature was 38°C. Conversion rates were obtained after integration of chromatograms and calculations to determine the percentages of transformation.

#### E1 and E1-S metabolism

From 17 to 35 mg of homogenized tissue from 12 paired samples was resuspended in 50 mM sodium phosphate buffer (pH 7.4), with protease inhibitors (one tablet of Complete™ [Roche Molecular Biochemicals, Basel, Switzerland]/10 mL buffer). The tissue suspensions were incubated with 10 nM estrone, [2,4,6,7-^3^H(N)]- and 6 mM NADPH in a reaction volume of 500 μL for 19 h at 37°C. The reaction was stopped with the addition of 100 μL 0.5 M ascorbic acid/1% acetic acid in methanol, and the mixture was centrifuged at 15,800 × g for 5 min. The supernatant was cleaned with solid phase extraction using Strata C-18E cartridges (Phenomenex, Aschaffenburg, Germany), according to the producer instructions, from which it was eluted with 2 × 350 μL methanol. The samples were then analyzed by HPLC (Beckman Coulter, Brea, CA, USA) using an Allure® Biphenyl Column [50 × 2.1 mm, particle size 3 μm (Restek Cooperation, Bellefonte, PA, USA)], with 55% (v/v) pure methanol mixed with 10% methanol, for 60 min at a flow rate of either 200 or 220 μL/min. Conversion rates were obtained after integration of chromatograms and evaluation with 24 Karat software (Beckman-Coulter, USA).

Additionally 24 mg of homogenized tissue from five paired samples was incubated with 16 nM ^3^H E1-S in 50 mM phosphate buffer, pH 7.4 using the same procedure as described above for A-dione.

#### Immunohistochemistry

Pairs of tissue microarrays (TMA) with 3 mm cores of tumor and adjacent control endometrium were prepared from formalin-fixed, paraffin embedded tissues from 44 hysterectomy specimens. Five micrometers thick TMA sections were prepared, de-waxed in xylene and rehydrated. Immunohistochemical stainings for HSD17B2 and STS were performed on a fully automated Ventana BenchMark GX System (Ventana Medical Systems, Inc., Tuscon, AZ, USA). Protocol included a pretreatment step using Cell Conditioning Solution 1 (48 min at 37°C; Tris-based buffer pH 8.5) and incubation in H_2_O_2_ to block endogenous peroxidase. The anti-HSD17B2 (Solvay Pharmaceuticals, 1:100/Ventana diluent) and anti-STS antibodies (donated from prof. Dr. Gerhard Schuler 1:2,000/Ventana diluent; Supplementary Table 3) were added manually and the primary antibody incubation was set for 2 h. The Optiview DAB Detection Kit was used according to the manufacturer instructions. Placenta was used as a positive control.

Staining for SULT1E1 and HSD17B1 was done manually with Novolink Polymer Detection System (Leica Biosystems, Wetzlar, Germany) according to manufacturer instructions. For HSD17B1 detection two different antibodies were used (Supplementary Table 3). Antigen retrieval was performed with Tris-EDTA buffer (pH 9) in a pressure cooker for 20 min and TMA sections were incubated with anti-HSD17B1 polyclonal and monoclonal antibodies (Solvay Pharmaceuticals, 1:4,000, 1 h, room temperature and ab51045 EP1682Y; Abcam; Cambridge, UK, 1:70 in 1% BSA/PBS, overnight, 4°C). For SULT1E1 (Supplementary Table 3) antigen retrieval was done in citrate buffer (pH 6) in a pressure cooker for 20 min. The TMA sections were incubated with anti-SULT1E1 antibodies (HPA028728, R28328, Sigma Aldrich, Germany, 1:100, overnight, 4°C). DAB chromogen solution was used to detect the bound antibodies.

The immunohistochemical staining was assessed by a pathologist (SFG) based on the staining intensity (scored as: 1, weak; 2, moderate; 3, strong) and the percentage of stained cells. The immunohistochemical scores were calculated by multiplying the percentage of positive cells (P) by the intensities (I) (SCORING = P × I; maximum = 300).

### Statistical evaluation

The differences in expression levels of the selected genes were analyzed at the mRNA and protein levels in the cancerous endometrium, as compared to the adjacent control endometrium, using Wilcoxon matched-pair tests. Depending on the normality of sample distribution, the steroid concentration measurements in cancerous and control tissue were evaluated with either paired *t*-test or Wilcoxon matched pairs test. The statistical tests were two-tailed. Stratification analyses were done using repeated measures ANOVA. The differences in *p*-values of <0.05 were considered to be significant. The statistical calculations and tests were performed using GraphPad Prism software, version 5.00 (San Diego, CA, USA).

## Results

### A-dione and E2 are present in both cancerous and adjacent control endometrium

We measured A-dione and E2 concentrations in 10 paired samples of EC and adjacent control endometrium using radioimmunoassay and chemiluminescent immunoassay, respectively, which are still in routine clinical application for measuring blood concentrations (Table 2). A-dione was detected in 9 out of 10 samples, and E2 in seven of these samples. The variability in A-dione and E2 levels between patients was large and we found no significant differences in A-dione and E2 levels (*p* = 0.193 and 0.375, respectively) between EC and adjacent control tissue.

**Table 2:** Androstenedione (A-dione) and estradiol (E2) concentrations in the cancer (Tumor) and adjacent control tissues (Control).

**Sample**	A-dione (pmol/g^*^)		E2 (pmol/g^*^)	
	**Control**	**Tumor**	**Control**	**Tumor**
49	0.63	0.41	2.24	3.47
50	5.08	6.54	13.00	4.71
51	<0.10	0.88	5.72	32.40
52	2.18	1.34	2.03	1.94
53	3.50	3.20	1.88	<0.40
54	17.19	9.22	3.76	3.06
55	1.69	0.30	<0.40	<0.40
56	0.20	<0.10	2.98	<0.40
57	5.27	1.45	11.73	7.67
58	1.53	1.34	0.78	0.97
Median	2.18	1.34	2.98	3.47
*p*-value	0.193		0.375	

* *pmol/g of powdered tissue*.

### A-dione is metabolized to T in cancerous and adjacent control endometrium

**Figure 2:**
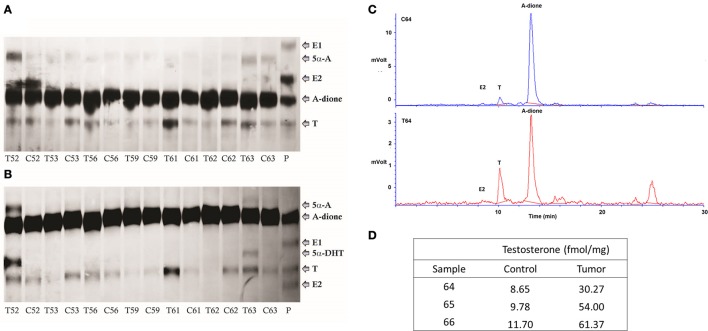
A-dione metabolism in the cancer and adjacent control endometrium. **(A)** Identification of the reaction products by autoradiography (TLC developed in cyclohexane/ ethyl acetate [1:1, v/v]) showing conversion of ^3^H labeled A-dione (80 nM) to testosterone (T) and 5α-androstanedione (5α-A) in cancer, T, and corresponding control, C, tissues. In placenta, P, A-dione was metabolized to T, E1, and E2. **(B)** Autoradiography of the same samples that were extracted from silica gel and developed in cyclohexane/ ethanol (95:5, v/v), confirming the presence of 5α-dihydrotestosteone (5α-DHT) in two EC samples. **(C)** HPLC profiles demonstrates metabolism of ^3^H labeled A-dione (8 nM) to T in control tissue and matched tumor tissue. **(D)** Formation of T (fmol/mg powdered tissue).

A-dione formed locally in EC or A-dione from circulation might serve as precursor for E2 formation. We thus examined the ability of EC tissue for aromatization. We studied the metabolism of 80 nM ^3^H labeled A-dione in seven paired samples of EC and adjacent control endometrium. In all of these samples, only conversion to T and much lower levels of 5α-androstanedione were detected by autoradiography after TLC in the first mobile phase (Figure 2A), while in the second mobile phase, 5α-dihydrotestosterone (5α-DHT) was also seen in two EC samples (Figure 2B). Aromatase activity was observed only in the control tissue, human placenta, where A-dione was metabolized to T, E1, and E2 (Figures 2A,B). As the first experiment included relatively high concentration of A-dione, we also examined the metabolism of 8 nM ^3^H labeled A-dione in the presence of the NADPH regeneration system in three paired samples of EC and adjacent control endometrium. In this experiment the products were separated by HPLC, where T was the major metabolite in all paired samples (Figure 2C) with increased formation of T seen in EC (Figure 2D). This is in agreement with our previous study in nine EC samples where 10 nM ^3^H labeled A-dione was metabolized mainly to T with no E2 seen (Vouk and Rizner, Unpublished data; Supplementary Figure 1).

### E1-S is metabolized to E2 in cancerous and adjacent control endometrium with increased formation of E2 seen in cancer tissue

**Figure 3:**
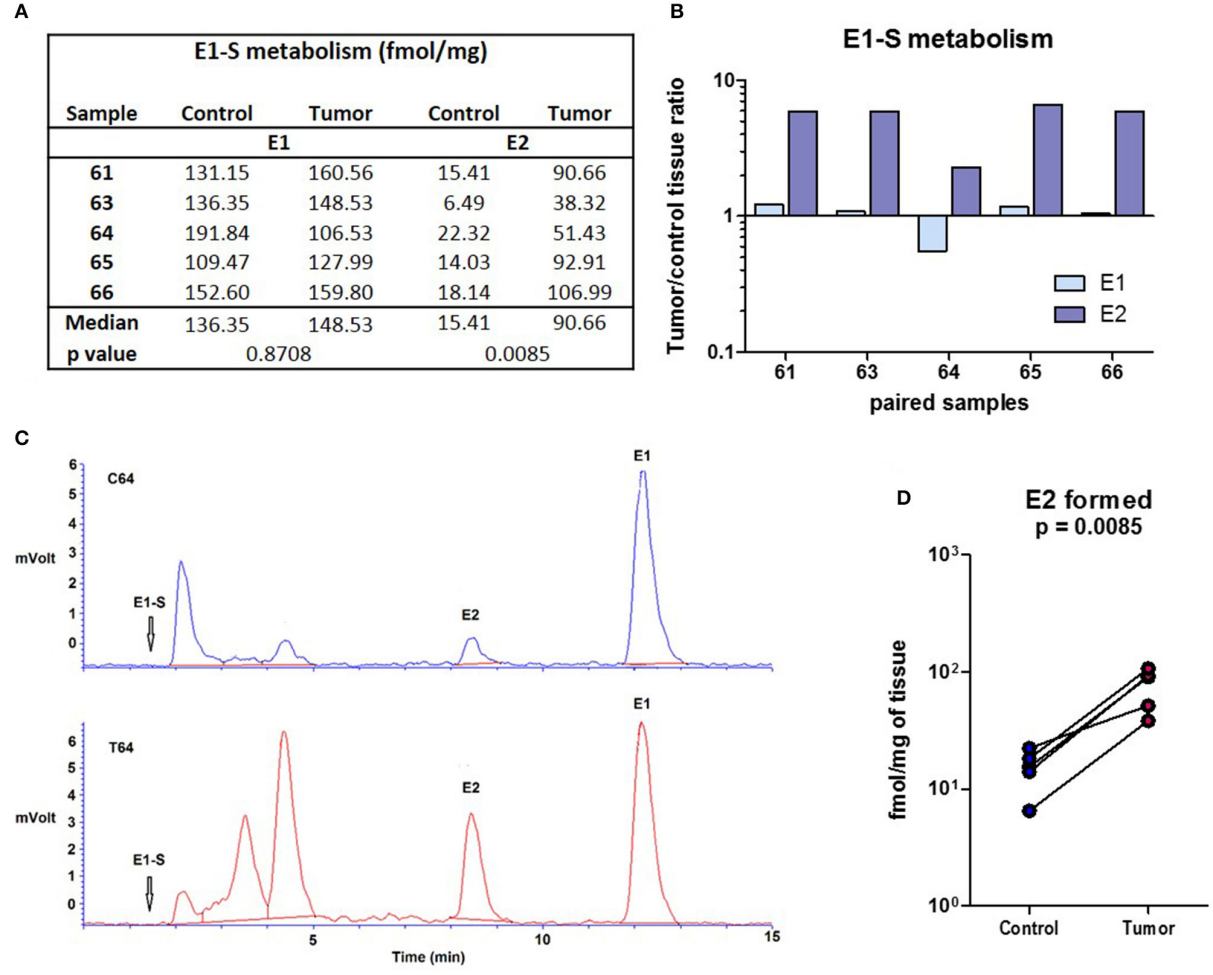
E1-S metabolism in the EC and adjacent control endometrium. **(A)** Table for the conversion of E1-S to E1 and E2 (fmol/mg powdered tissue) in control and corresponding EC. **(B)** Histogram with logarithmic scale demonstrates increased tumor/ control tissue ratio for E2 formation in all paired samples. **(C)** HPLC elution profiles shows the formation of E2 and E1 from ^3^H labeled E1-S (16 nM) for the representative samples of control (C64) and corresponding cancer (T64) tissues. Rf of E1-S and formation of several unidentified polar metabolites is also shown. **(D)** Before-and-after graph demonstrates levels of E2 formed in control endometrium (Control) and the corresponding EC (Tumor) for samples 61, 63, 64, 65, and 66.

**Figure 4:**
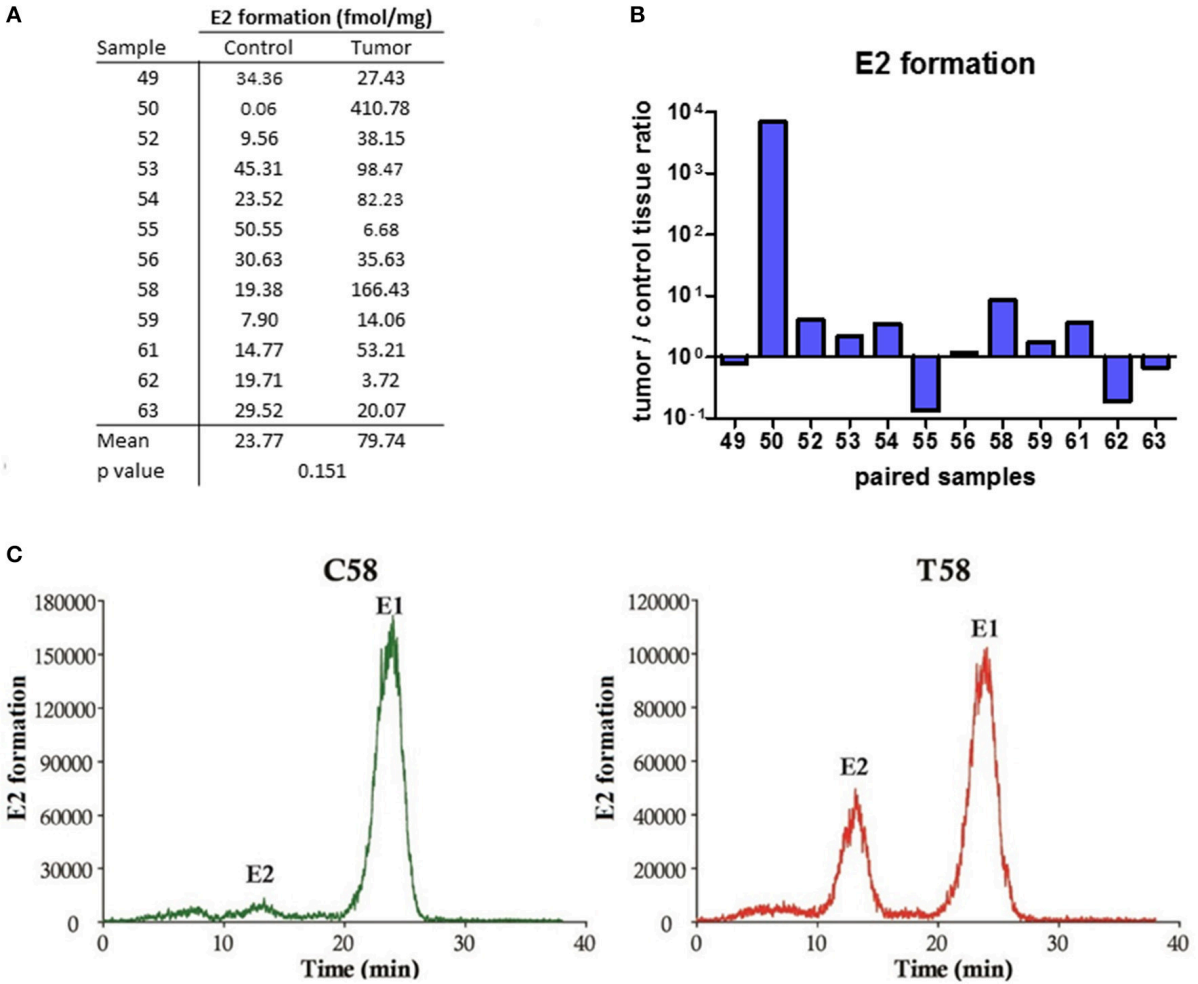
E2 formation in the EC and adjacent control tissues. **(A)** Table showing the formation of E2 (fmol/mg powdered tissue) in control and corresponding cancer endometrium. **(B)** Histogram with logarithmic scale demonstrates increased tumor/ control tissue ratio for E2 in 7 of 12 paired samples. **(C)** HPLC elution profile shows the formation of E2 from E1 for representative control (C58) and cancer (T58) tissues.

Since no E2 were formed by metabolism of A-dione in EC specimens, we next studied the ability of this tissue for metabolism of the major circulating estrogen, E1-S. This study was performed in five paired samples of EC and adjacent control tissue. Sixteen nM E1-S was metabolized to E1 and E2 in both, EC and adjacent control tissue, with significantly higher levels of E2 formed in cancerous tissue (*p* = 0.0085; Figures 3A,B,D). In addition to E2 and E1 also several unidentified polar metabolites were formed (Figure 3C). The metabolism of 10 nM E1 was further examined in 12 paired samples of EC and adjacent control endometrium. The formation of E2 was detected in all of the samples (Figures 4A–C); with a 2-fold, but non-significantly increased median levels seen in cancerous tissue (*p* = 0.151; Figure 4A). In seven samples of EC there was an increased E2 formation, in one sample there was no difference and in four samples of EC originating from one patient with serous EC and three patients with well-differentiated EC (G1) there was a decreased E2 formation as compared to adjacent control endometrial tissue (Figure 4A).

### Genes for local formation of E2 via the aromatase and sulfatase pathways are expressed in cancerous endometrium

**Figure 5:**
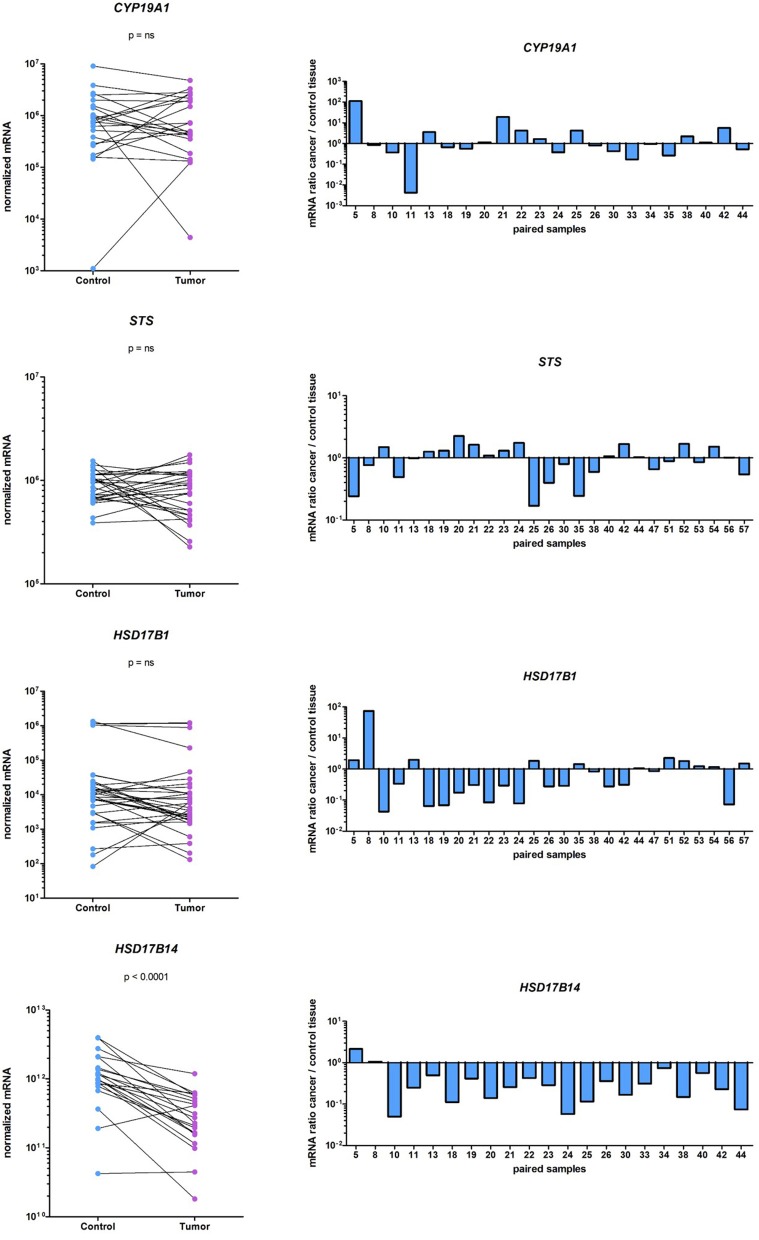
Expression of *CYP19A1*, *STS*, *HSD17B1*, and *HSD17B14* in the EC and adjacent control tissues. On the left before-and-after graphs show the normalized expression levels of the genes investigated in control endometrium (Control) and the corresponding cancer endometrium (Tumor). The levels of gene expression are presented on a logarithmic scale. On the right are histograms with logarithmic scale shows mRNA ratio between cancer and control tissue.

Genes that are involved in local E2 formation from A-dione or E1S (Figure 1) are expressed in cancerous and adjacent control tissue. In the same cohort of EC patients, we previously reported no statistically significant differences in the expression of the majority of the genes involved in E2 formation: *CYP19A1, STS, HSD17B4, HSD17B8, HSD17B12*, and *SULT1E1*; however, *HSD17B1*, and *HSD17B7*, which promote E1 activation to E2, were down-regulated, and *HSD17B2*, which has the opposite role, was upregulated in the EC samples (Smuc and Rizner, 2009; Sinreih et al., 2013). In the present study, we re-examined the mRNA levels of *CYP19A1, STS*, and *HSD17B1* on a larger cohort of samples and found very low and unaltered mRNA levels of *CYP19A1* and *HSD17B1*. The mRNA levels of *STS* were about 1,000-fold higher than the levels of *HSD17B1*, but still unchanged in the EC and control tissues (Figure 5). With an arbitrary threshold of 1.2 for the ratio of mRNA levels in pairs of EC and adjacent control tissue we saw increased ratios in 8 out of 22 pairs for *CYP19A1*, 10 out of 27 pairs for *STS* and 9 out of 27 pairs for *HSD17B1*. We also investigated the mRNA levels of the oxidative *HSD17B14*, which has not yet been studied in EC and found high, but statistically significantly decreased levels (*p* < 0.0001) in cancer tissue, compared to control tissue.

Further, stratification according to clinical data (menopausal status and vital status of the patients' and FIGO stage) and histopathological data (histological type and grade of the tumor, depth of myometrial invasion, and presence of lymphovascular invasion) revealed differences in *STS, SULT1E1*, and *HSD17B14* expression (Table 3). *STS* was significantly downregulated (*p* = 0.0439) only in high grade tumors (G3) while in lower grade tumors (G1 and G2) *STS* levels did not differ between cancer and adjacent control tissue. The expression of *SULT1E1* was significantly downregulated (*p* = 0.0392) in cancer tissue from premenopausal women, with significantly lower levels seen in cancer and adjacent control tissue from postmenopausal women as compared to premenopausal women. The expression of *HSD17B14* changed in more invasive cancers, there was an extensive downregulation in cancer compared to adjacent control tissue form patients with lymphovascular invasion (*p* = 0.0298).

**Table 3:** Changes in expression of genes according to histopathological and clinical characteristics of endometrial cancer patients.

**Gene**	**Tumor/control**		**Tumor differentiation (high grade vs. low grade)**	**Menopausal status**	**FIGO stage (IA vs. IB-IV)**	**Myometrial invasion (yes/no)**	**Lymphovascular invasion (yes/no)**	**Vital status**
	**Fold-change**	***p***	***p***	***p***	***p***	***p***	***p***	***p***
*CYP19A1*	0.85	0.5203	0.1115	0.0615	0.0893	0.1830	0.1995	0.3106
*STS*	0.93	0.7639	**0.0439**	0.0868	0.4326	0.7682	0.3991	0.0529
*HSD17B1*	0.75	0.2110	0.1562	0.9748	0.6552	0.1273	0.1844	0.3882
*HSD17B14*	0.26	**<0.0001**	0.3136	0.2847	0.8550	0.4597	**0.0298**	0.4233
*SULT1E1*	0.43	0.1606	0.2128	**0.0392**	0.2615	0.7745	0.2252	0.3653

*The differences in expression levels in tumor as compared to the adjacent control endometrium were analyzed using Wilcoxon matched-pair tests. Stratification analysis were done using 2-way ANOVA. The differences in p-values of < 0.05 were considered significant and are marked in bold*.

### High protein levels of STS and HSD17B2 are seen in cancerous and adjacent control endometrium

**Figure 6:**
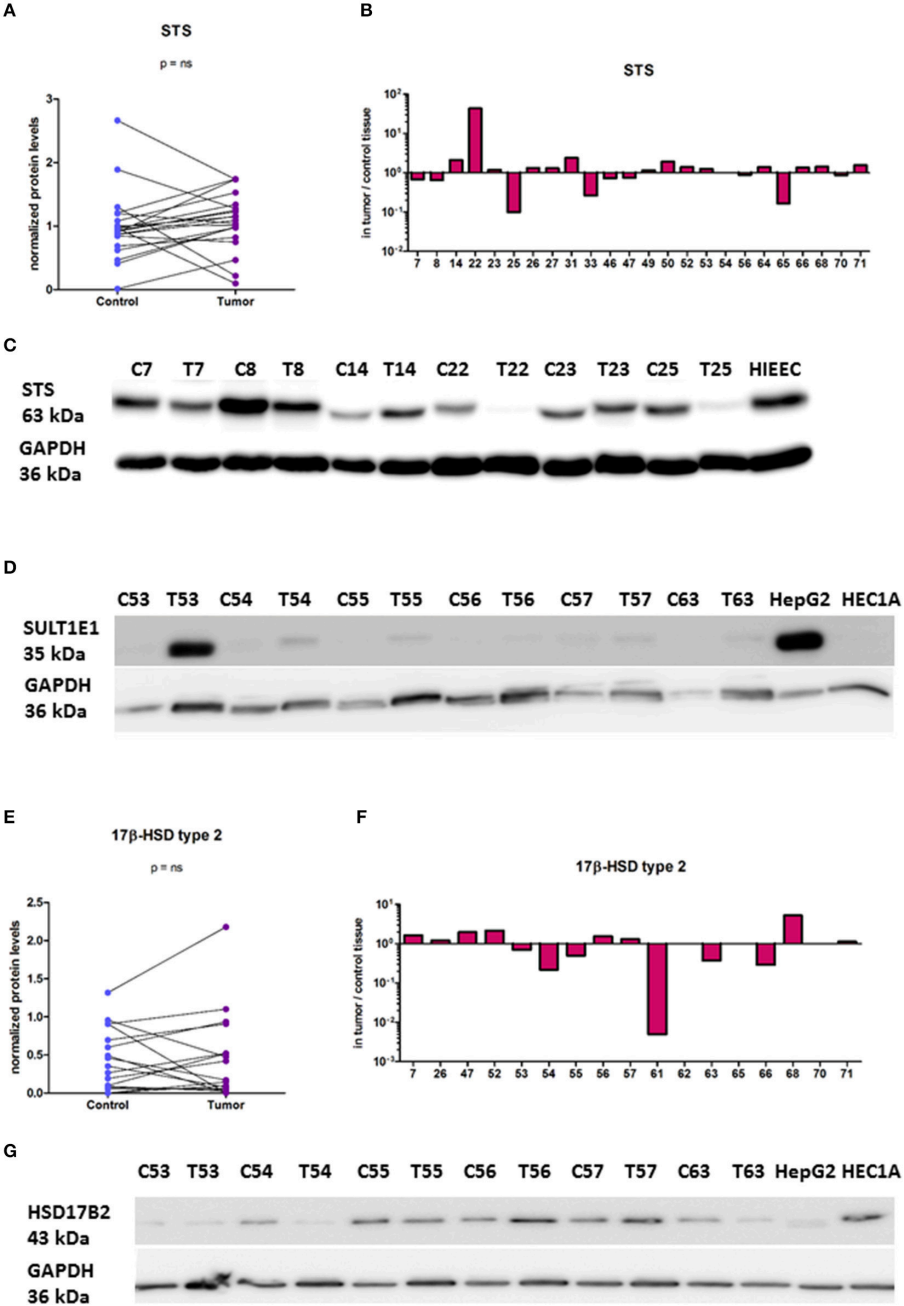
Protein levels of STS, SULT1E1, and HSD17B2 in the EC and adjacent control tissue. **(A)** Before-and-after graph shows STS protein levels in 24 paired samples of control endometrium (Control) and corresponding cancer tissue (Tumor). The data was quantified and normalized to GAPDH levels. **(B)** Histogram with logarithmic scale demonstrates higher STS levels in cancer endometrium in 12 paired samples out of 24. **(C)** Representative membrane with STS and GAPDH staining. **(D)** Representative membrane with SULT1E1and GAPDH staining. **(E)** Before-and-after graph shows HSD17B2 protein levels in 17 paired samples of control endometrium (Control) and corresponding cancer tissue (Tumor). The data was quantified and normalized to GAPDH levels. **(F)** Histogram with logarithmic scale demonstrates protein ratio between paired samples of tumor and control tissue. **(G)** Representative membrane with HSD17B2 and GAPDH staining. C, control endometrium; T, EC tissue; HIEEC, control epithelial cell line of normal endometrium; HEC1A, endometrial cancer cell line; HepG2, liver cancer cell line.

As mRNA levels do not necessary correlate with protein levels and enzymatic activity, we also examined protein levels of STS, SULT1E1, HSD17B1, and HSD17B2. With the specific antibodies we performed Western blot analysis to evaluate protein levels of these enzymes in paired samples (Figure 6). We found high protein levels of STS, with increased levels in 12 EC samples out of 24, where this difference was not statistically significant (Figures 6A–C). SULT1E1 protein levels were very low in all but one tumor sample (T53, Figure 6D) thus it was not possible to accurately estimate differences in protein levels between control compared to EC tissue. We were not able to detect HSD17B1 protein in EC tissues using two different antibodies (rabbit monoclonal antibody, EP1682Y, Abcam UK; and rabbit polyclonal antibodies from Solvay Pharmaceuticals; data not shown) although these antibodies recognized HSD17B1 in placenta tissue and in homogenates of *E. coli* overexpressing *HSD17B1*. Protein levels of HSD17B2 were seen in the majority of samples, with increased levels in 7 out of 17 pairs, but with no statistically significant difference between EC and adjacent control tissue (Figures 6E–G). The stratification according to clinical and histopathological data confirmed the effects of tumor differentiation on STS expression (Table 4). The significantly lower STS protein (*p* = 0.0039) levels in EC as compared to adjacent control tissue were seen only in high grade tumors (G3), while in well-differentiated tumors (G1, G2) there were no differences in STS levels between EC and control tissue (Table 4). However, this trend was not supported by further immunohistochemical staining.

**Table 4:** Changes in protein levels according to histopathological and clinical characteristics of endometrial cancer patients.

**Protein**	**Tumor/control**		**Histological differentiation (high grade vs. low grade)**	**Menopausal status**	**FIGO stage (IA vs. IB-IV)**	**Myometrial invasion (yes/no)**	**Lympho-vascular invasion (yes/no)**	**Vital status**
	**Fold-change**	***p***	***p***	***p***	***P***	***p***	***p***	***p***
STS	1.09	0.2660	**0.0039**	0.9142	0.1013	0.7690	0.5050	0.5068
HSD17B2	1.03	0.8498	0.2891	0.0989	0.3028	0.5704	0.6941	0.6856

*Protein levels were determined by Western blotting. The differences in expression levels in tumor as compared to the adjacent control endometrium were analyzed using Wilcoxon matched-pair tests. Stratification analysis were done using 2-way ANOVA. The differences in p-values of < 0.05 were considered significant and are marked in bold*.

**Figure 7:**
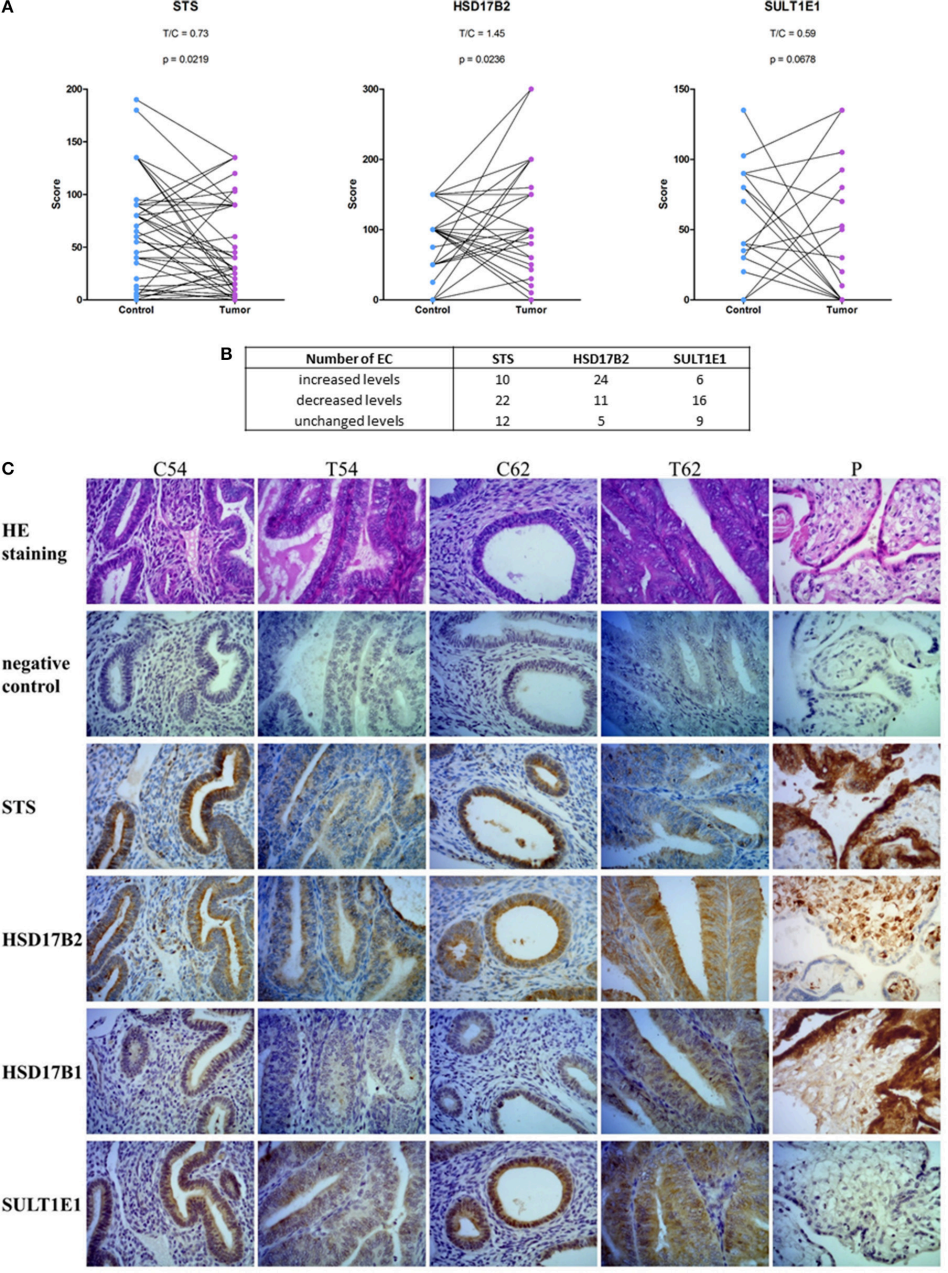
Immunohistochemical staining for STS, HSD17B2, HSD17B1, and SULT1E1 in endometrial cancer. **(A)** Before-and-after graphs show scoring for STS, HSD17B2, and SULT1E1 in control endometrium (Control) and the corresponding cancer endometrium (Tumor). **(B)** Table with number of samples with increased, decreased or unchanged levels of protein in tumor tissue compared to adjacent control endometrium. **(C)** representative staining of adjacent control endometrium (C) and cancerous tissue (T) for STS, HSD17B2, HSD17B1, and SULT1E1 (samples 54 and 62), and placenta (P) as a positive control. Samples were stained with hematoxylin and eosin (HE staining) and with specific antibodies against STS, HSD17B2, HSD17B1, and SULT1E1. 400 × magnifications are shown.

With the same set of antibodies as previously used for Western blotting we performed immunohistochemical staining of tissue microarrays, which included 44 pairs of cancer and adjacent control tissue. We observed staining for STS, HSD17B2, HSD17B1, and SULT1E1 in EC and adjacent control tissue (Figures 7A,C). STS staining indicated clear cytoplasmic reaction with several samples showing distinct luminal accumulation of this protein. Scoring and further statistical analysis revealed overall significantly lower levels of STS (*p* = 0.0219) in cancer compared to adjacent control tissue but with unchanged levels in 12 pairs, increased levels in 10 pairs and decreased levels in 22 out of 44 pairs (Figures 7A,B, Supplementary Table 4). HSD17B2 showed granulated cytoplasmic reaction in all samples. Protein levels of HSD17B2 were in general significantly increased (*p* = 0.0236) in cancer as compared to adjacent control tissue with unchanged levels in 5 pairs, decreased levels in 11 pairs, and increased levels in 24 out of 40 pairs (Figures 7A,B, Supplementary Table 4). Staining for HSD17B1 with Abcam EP1682Y antibodies was weak but indicated distinct and clear cytoplasmic reaction with clearly negative control staining and intense staining in placenta tissue, which served as a positive control (Figure 7C). Weak staining for HSD17B1 was seen in 38 control and 36 cancer samples out of 42 pairs investigated. With Solvay antibodies against HSD17B1 moderate staining was seen in control and cancer tissue in epithelial and stromal cells, with cytoplasmic but also some positive nuclear staining, and intense staining in placenta tissue (data not shown). Staining for SULT1E1 in EC and adjacent control endometrial tissue samples was cytoplasmic with no significant difference between cancer and the adjacent control tissue, but with decreased levels in 16 pairs, increased levels in six pairs and no staining in 8 out of 31 pairs (Figures 7A,B; Supplementary Table 4). The same antibody intensively stained small intestine and duodenum tissue, which served as positive controls and weakly lung tissue, which was a negative control (data not shown). Stratification of the experimental data according to clinical and histopathological characteristics of patients revealed no effects on STS, HSD17B2, and SULT1E1 levels (Table 5).

**Table 5:** Changes in immunohistochemical scoring according to histopathological and clinical characteristics of endometrial cancer patients.

**Protein**	**Tumor/control**		**Tumor differentiation (high grade vs. low grade)**	**Menopausal status**	**FIGO stage (IA vs. IB-IV)**	**Myometrial invasion (yes/no)**	**Lympho-vascular invasion (yes/no)**	**Vital status**
	**Fold-change**	***p***	***p***	***p***	***p***	***p***	***p***	***p***
STS	0.73	**0.0219**	0.6383	0.8962	0.8787	0.5752	0.693	0.7503
HSD17B2	1.45	**0.0236**	0.5055	0.5419	0.9576	0.45	0.7844	0.4178
SULT1E1	0.59	0.0678	0.657	0.0535	0.8447	0.6497	0.1287	0.3016

*The differences in expression levels in tumor as compared to the adjacent control endometrium were analyzed using Wilcoxon matched-pair tests. Stratification analysis were done using 2-way ANOVA. The differences in p-values of < 0.05 were considered significant and are marked in bold*.

## Discussion

In postmenopausal EC patients estrogens can be formed in peripheral tissues, from inactive precursors of adrenal (DHEAS, DHEA) or ovarian origin (A-dione), or from circulating E1-S. However, the local formation of E2 in cancerous endometrium and especially the contributions of the sulfatase and aromatase pathways has not been clearly defined which called for further studies. We first measured concentrations of A-dione, which can serve as a precursor for E2 biosynthesis via the aromatase pathway and E2 itself in paired samples of EC and adjacent control endometrium. A-dione and E2 were detected almost in all samples, but with large variability between patients, similarly as reported previously for the measurements of mammary E2 (Chetrite et al., 2000; Geisler et al., 2000). Previously Berstein et al. (2003) detected significantly higher E2 levels by radioimmunological assay in 78 cases of cancer tissue compared to the macroscopically normal adjacent endometrium (mean levels 0.498 and 0.314 pmol/g wet tissue, respectively). We calculated the hormone levels per mass of powdered tissue and thus determined about 10-fold higher concentrations. Both studies used immunological assays which introduced certain methodological limitations. In the last decade the reliability of immunoassays has been questioned due to the potential cross-reactivity between different stereoisomers and low sensitivity, thus GC/MS and LC-MS/MS techniques are now recommended (Penning et al., 2010; Stanczyk and Clarke, 2010). To the best of our knowledge these methodologies have not yet been used for determination of A-dione and E2 concentrations in EC tissue. Although, the immunological assays used in this study might have not determined exact concentrations of A-dione and E2, they have confirmed presence of A-dione and E2 in the majority of cancer and adjacent control tissues, which supports local formation of E2.

Detection of A-dione in cancerous endometrium led us to examine its metabolism in paired samples of EC and adjacent control tissue. Our data show that A-dione at 80, 10, and 8 nM is reduced to T where no E2 is formed. These results were confirmed, altogether in 19 EC samples, by two methodological approaches (TLC with autoradiography and HPLC separations using radioactivity detector) also in the presence of regeneration system which provided sufficient levels of NADPH. Reduction of A-dione to T can be catalyzed by the reductive androgenic 17β-HSD type 5 (HSD17B5), better known as AKR1C3, which was shown to be expressed in EC samples from our cohort (Smuc and Rizner, 2009). The A-dione metabolism data using physiological steroid concentrations suggests a marginal role for aromatase in EC tissues, which is in agreement with the reports of Fournier and Poirier and our previous studies showing no E1 and E2 formation in four EC cell lines (Ishikawa, HEC-1A, HEC-1B and RL-95) after 24 h incubation with 8 nM A-dione (2009) (Fournier and Poirier, 2009; Hevir-Kene and Rižner, 2015).

As our data show that E2 cannot be formed from A-dione via the aromatase pathway, we next studied formation from E1-S via the sulfatase pathway. The E1-S and E1 metabolism studies in 5 and 12 paired samples of EC and adjacent control endometrium, respectively, support formation of E2 from both precursors with higher levels of E2 in EC. This data thus confirm that in EC the most potent estrogen E2 can be formed via the sulfatase pathway from circulating E1-S as also supported by increased E1-S plasma levels in EC patients compared to healthy postmenopausal women (Lepine et al., 2010; Audet-Walsh et al., 2011; Brinton et al., 2016). Our E1-S metabolism data also show significantly increased levels of E2 in cancer tissue Similarly, Cornel et al., reported that reduction of E1 to E2 predominates over oxidation of E2 to E1 in EC compared to normal endometrial tissue and adjacent control tissue (Cornel et al., 2012).

The expression of the majority of these genes for local E2 formation via the aromatase and the sulfatase pathways has previously been studied in the same cohort of patients (Smuc and Rizner, 2009). Here, we re-examined expression of genes encoding the key enzymes of the aromatase and the sulfatase pathway, *CYP19A1, STS*, and *HSD17B1* on a larger number of samples. The very low and unchanged *CYP19A1* expression also reported by Cornel et al. (2012) support our A-dione metabolism data, which shows that aromatization of androgens is not the primary mechanism for E2 formation in EC.

On the other hand, high but unchanged expression of *STS* at the mRNA level, unchanged protein levels of STS in EC samples seen by Western blotting and significantly lower protein levels seen by IHC, imply that STS may have more important role in the adjacent control tissue as compared to EC. However, statistical analysis revealed that *STS* is downregulated in high grade cancers but not in low grade cancers, where similar levels were seen in EC and the adjacent control tissue (Table 3). Similar trend was seen also for protein levels of STS as determined by Western blotting (Table 4). However, due to a low number of high grade cancers, this association have to be considered with caution and the effect of high grade cancer should be reassessed on a higher number of samples.

The importance of the sulfatase pathway is also supported by the previously reported 5-fold lower expression of *SULT1E1* as compared to *STS* and decreased mRNA levels of *SULT1E1* in 27 out of 38 EC samples from the same cohort, where this difference did not reach statistical significance (Hevir et al., 2011b) and also by significantly increased ratio between *STS* and *SULT1E1* in EC. Furthermore, our current statistical analysis, showed significantly lower *SULT1E1* mRNA levels in EC samples from premenopausal women and several fold downregulated *SULT1E1* expression in postmenopausal patients with no effects of tumor differentiation (Table 3). The protein levels of SULT1E1 were very low, which is in line with mRNA levels, reports of others (Utsunomiya et al., 2004) and Human Protein Atlas (data obtained on April 28, 2017). Additionally, IHC staining for SULT1E1 was negative (>25% cases) or weak with a clear trend for decreased levels in EC (Supplementary Table 4). This data thus support the capacity of EC tissue for activation of E1-S to E1.

HSD17B1 is the most obvious candidate for reduction of E1 to E2. In this study, we saw low, but statistically unchanged mRNA levels of *HSD17B1* in EC as compared to control adjacent tissue. In 9 patients out of 27 these mRNA levels were increased in EC compared to adjacent control tissue. The gene expression of other reductive enzymes, as HSD17B7 and HSD17B12, was previously seen to be decreased and unchanged in the same cohort of EC, respectively (Smuc and Rizner, 2009). The average expression levels of *HSD17B7* were 5-fold higher than *HSD17B1*, while *HSD17B12* levels exceeded those of *HSD17B1* by more than 10^4^-fold (Smuc and Rizner, 2009), implying that also HSD17B12 is important for E1 activation. Additionally, AKR1C3, which preferentially catalyzes the reduction of A-dione, might contribute to E2 formation, as shown after its overexpression in the MCF7 breast cancer cell line (Byrns et al., 2010). At the protein level we saw a weak specific cytoplasmic IHC staining for HSD17B1 in EC and control endometrial epithelial cells with no difference between cancer and adjacent control tissue. At the protein level Cornel et al., previously reported (Cornel et al., 2012) increased immunoreactivity in grade 1 EC and unchanged levels in high grade EC, whereas in their most recent study they reported very weak staining for HSD17B1 in EC (Cornel et al., 2016). Our results are in line with a low *HSD17B1* expression levels observed in cancer and adjacent control tissue. Due to the higher catalytic efficiency of HSD17B1 as compared to the other isoforms, low expression levels might still result in high conversion of E1 to E2 (Gangloff et al., 2001). Higher capacity of HSD17B1 for E2 formation, compared to other enzymes, was also confirmed by transient transfection of HSD17B1, HSD1712, and AKR1C3 in EC cell line ECC1 (Cornel et al., 2012).

HSD17B2 has the highest catalytic efficiency for oxidation of E2 to E1 and we recently reported significantly increased mRNA levels in EC compared to adjacent control tissue, where pairwise comparison showed increased levels in 32 EC samples out of 47 pairs (Sinreih et al., 2013). Our current Western blotting showed increased protein levels of HSD17B2 in cancer endometrium in 7 out of 17 paired samples while IHC revealed significantly increased protein levels in EC samples compared to adjacent control tissue but with lower levels seen in 11 EC samples out of 40 investigated (Supplementary Table 4). Among other oxidative HSD17Bs with lower catalytic efficiencies, we previously saw unchanged mRNA levels of *HSD17B4* and *HSD17B8* (Smuc and Rizner, 2009). In this study, *HSD17B14* was in general significantly downregulated at the mRNA level and we saw lower expression in 19 out of 21 pairs of EC and adjacent control endometrium. Interestingly, more extensive downregulation of *HSD17B14* was seen in EC samples from patients with lymphovascular invasion (Table 3). Oxidation of E2 to E1 can be catalyzed by several isozymes. Although, HSD17B2 has the highest catalytic efficiency for oxidation of E2 to E1 also decreased expression of *HSD17B14* gene encoding the oxidative enzyme HSD17B14 may affect E2 levels.

The increased formation of E2 seen in EC certainly results from the disturbed balance between the reductive and oxidative isoforms of HSD17Bs. However, the unchanged mRNA levels of *HSD17B1* and *HSD17B12*, increased mRNA levels of *HSD17B2* and decreased mRNA levels of *HSD17B14* in EC do not fully explain higher capacity for E2 formation seen in EC tissue. Our results thus support studies in model cell lines, where individual inhibitors of HSD17B1, HSD17B5 (AKR1C3), HSD17B7, and HSD17B12 failed to completely block E2 formation (Fournier and Poirier, 2009). Our experimental data indicate that HSD17B1 might not be solely accountable for E2 formation and suggest that large differences in catalytic efficiencies of the reductive estrogenic HSD17B enzymes might partially be compensated by higher expression levels of other isozymes and also higher local concentration of E1 in EC tissue.

The local concentration of E1 and E2 depends on concentration of E1-S and especially activity of STS, which is highly expressed in EC tissue with unchanged protein levels in low grade cancers compared to adjacent control tissue, and is only weakly opposed by *SULT1E1*. The sulfatase pathway is thus clearly implicated in local estrogen formation, and concomitant enhanced estrogen actions in EC. It has to be stressed here that a great variabilities in gene expression at the mRNA and protein levels have been seen among patients. Also increased capacity for E2 formation in cancerous endometrium has not been observed in all patients.

STS inhibitors have been considered as novel anticancer agents where phase II clinical study has already been performed in ER-positive advanced/recurrent EC, however with no convincing results for STS inhibitor irosustat vs. progestin megestrol acetate (Pautier et al., 2017) with the progression free survival of 16 and 32 weeks, respectively. STS inhibitor thus performed worse as compared to the current medical treatment for recurrent EC. This suboptimal performance of STS inhibitor is not in contrast with the reported increased E2 formation in cancerous endometrium. Our cohort included EC patients with mostly well-differentiated low grade primary cancer. The situation in high grade cancers and especially advanced/recurrent cases may be different as suggested by decreased *STS* mRNA and protein levels seen in high grade vs. low grade cancers. It is clear that further studies including high grade and advanced cancers and also focusing on E1S uptake transporters are needed to clarify the clinical performance of STS inhibitor.

Altogether our study confirms the presence of E2 in cancerous endometrium, the capacity of this tissue for activation of E1S to E2 and reports expression of genes involved in local E2 formation via the sulfatase pathway at the mRNA and protein levels. This data is extended by a statistical evaluation of individual variables including histopathological and clinical characteristics that may affect the expression levels. Although the limitation of our study is a relatively low number of samples included in the individual analyses, the findings on the local E2 formation are supported by different methodological approaches and are substantiated by the protein levels and enzymatic activities of STS and HSD17B1 in EC tissue.

Formation of E2 in EC also depends on intracellular E1-S concentrations, which are regulated by the availability of Organic Anion Transporting Polypeptides (OATPs) and Organic Anion Transporters (OATs), where several OATPs and OATs catalyze the cellular uptake of E1-S (Mueller et al., 2015). However, the expression of genes encoding OATP and OAT transporters has not yet been examined in EC. As the concerted action of these transporters and intracellular enzymes is required for local E2 formation this lack of knowledge currently precludes the complete understanding of E2 formation in cancerous endometrium and calls for further studies. Especially, as these transporters may have crucial roles in local estrogen formation and may represent novel options for treatment.

## Author contributions

TLR designed the study, contributed to writing of the manuscript and provided critical assessment and final approval of the manuscript. MS, TK, NH, MA, KV, and AJ carried out the experimental work, analyzed the results, and contributed to writing of the manuscript. SF assessed immunohistochemical staining, contributed to writing of the manuscript, and provided critical assessment of the manuscript.

### Conflict of interest statement

The authors declare that the research was conducted in the absence of any commercial or financial relationships that could be construed as a potential conflict of interest.

## Abbreviations

A-dione: androstenedione
AKR: aldo-keto reductase
DHEA: dehydroepiandrosterone
DHEA-S: dehydroepiandrosterone sulfate
EC: endometrial cancer
E1: estrone
E1-S: estrone sulfate
E2: estradiol
ER: estrogen receptor
HSD: hydroxysteroid dehydrogenase
qPCR: quantitative real-time PCR
STS: sulfatase
SULT: sulfotransferase
T: testosterone.
