# Supplementary material for: The Significance of the Sulfatase Pathway for Local Estrogen Formation in Endometrial Cancer
Source: Front Pharmacol. 2017 Jun 23;8:368. doi: 10.3389/fphar.2017.00368 (PMC5481366; doi:10.3389/fphar.2017.00368)
Supplement: Supplementary file 1 [file Table1.docx]

Supplementary Materials

**The significance of the sulfatase pathway for local estrogen formation in endometrial cancer**

Maša Sinreih, Tamara Knific, Maja Anko, Neli Hevir, Katja Vouk, Aleš Jerin, Snježana Frković Grazio and Tea Lanišnik Rižner^*^

^1^Institute of Biochemistry, Faculty of Medicine, University of Ljubljana, Slovenia

^2^Institute of Clinical Chemistry and Biochemistry, University Medical Centre, Ljubljana, Slovenia

^3^Department of Pathology, Division of Obstetrics and Gynecology, University Medical Centre, Ljubljana, Slovenia

*Address all correspondence and reprints requests to:

Dr. Tea Lanišnik Rižner

Institute of Biochemistry

Faculty of Medicine

University of Ljubljana

Vrazov trg 2

1000 Ljubljana

Slovenia

Tel: +386-1-5437657

Fax: +386-1-5437641

E-mail: [Tea.Lanisnik-Rizner@mf.uni-lj.si](mailto:Tea.Lanisnik-Rizner@mf.uni-lj.si)

**Supplementary Table 1 Histopathological and clinical data of endometrial cancer patients.**

| **Number** | **Sample** | **Age** | **Histological type** | **Depth of myometrial invasion** | **Presence of lymphovascular invasion** | **FIGO stage** | **Menopausal status** | **BMI** | **Method (1=qPCR, 2=IHC, 3=WB, 4=TLC, 5=HPLC, 6=steroid concentrations)** |
| --- | --- | --- | --- | --- | --- | --- | --- | --- | --- |
| 1 | **1** | 52 | G1 | < 50% | no | IA | premenopausal | 24.2 | 2 |
| 2 | **3** | 65 | G1 | < 50% | no | IA | postmenopausal | 33.6 | 2 |
| 3 | **4** | 50 | serous | < 50% | no | IA | postmenopausal | 27.9 | 2 |
| 4 | **5** | 39 | dedifferentiated | > 50% | yes | IB | premenopausal | 21.7 | 1 |
| 5 | **6** | 76 | serous | > 50% | yes | IIIC1 | postmenopausal | 23.9 | 2 |
| 6 | **7** | 50 | G1 |  |  | IB | premenopausal |  | 3 |
| 7 | **8** | 83 | dedifferentiated | > 50% | no | IA | postmenopausal |  | 1,3 |
| 8 | **9** | 41 | G1 | < 50% | no | IA | premenopausal | 46.1 | 2 |
| 9 | **10** | 53 | G1 | < 50% | no | IA | postmenopausal | 28.3 | 1,2 |
| 10 | **11** | 60 | G2 | > 50% | yes | IB | postmenopausal | 25.0 | 1,2 |
| 11 | **13** | 64 | G1 | < 50% |  | IV | postmenopausal | 26.2 | 1 |
| 12 | **14** | 73 | G1 | > 50% | no | IB | postmenopausal | 34,1 | 3 |
| 13 | **18** | 79 | G1 | > 50% | no | IB | postmenopausal | 32.8 | 1 |
| 14 | **19** | 74 | G1 | < 50% | no | IA | postmenopausal | 28.6 | 1,2 |
| 15 | **20** | 76 | G1 | < 50% | no | IA | postmenopausal | 32.4 | 1 |
| 16 | **21** | 53 | G2 | < 50% | no | IA | premenopausal | 27.3 | 1,2 |
| 17 | **22** | 36 | G1 | < 50% | no | IA | premenopausal | 33.8 | 1,2,3 |
| 18 | **23** | 45 | G1 | < 50% | no | IA | premenopausal | 20.0 | 1,2,3 |
| 19 | **24** | 69 | G2 | > 50% | yes | IB | postmenopausal | 25.3 | 1,2 |
| 20 | **25** | 54 | G3 | < 50% | no | IA | premenopausal | 23.0 | 1,2,3 |
| 21 | **26** | 72 | G1 | < 50% | no | IA | postmenopausal | 35.9 | 1,2,3 |
| 22 | **30** | 54 | G1 | < 50% | no | IA | premenopausal | 19.9 | 1,2 |
| 23 | **31** | 69 | G3 | > 50% | yes | IB | postmenopausal | 30.1 | 1,2,3 |
| 24 | **33** | 77 | G3 | > 50% | no | IB | postmenopausal |  | 1,3 |
| 25 | **34** | 57 | G1 | < 50% | no | IA | postmenopausal | 38.2 | 1,2 |
| 26 | **35** | 61 | G1 | < 50% | no | IA | postmenopausal | 30.8 | 1,2 |
| 27 | **38** | 78 | G2 | < 50% | yes | IA | postmenopausal |  | 1,2 |
| 28 | **40** | 71 | serous | < 50% | no | IA | postmenopausal | 29.4 | 1,2 |
| 29 | **42** | 81 | G1 | < 50% | no | IA | postmenopausal | 28.4 | 1,2 |
| 30 | **44** | 73 | serous | > 50% | yes | IB | postmenopausal | 24.8 | 1,2 |
| 31 | **46** | 50 | G2 | < 50% | no | IIIA | premenopausal | 32.3 | 1,2,3 |
| 32 | **47** | 27 | dedifferentiated | < 50% | no | IA | premenopausal | 20.0 | 1,2,3 |
| 33 | **49** | 70 | G1 | < 50% | no | IA | postmenopausal | 47.7 | 2,3,5,6 |
| 34 | **50** | 73 | G1 | < 50% | no | IA | postmenopausal | 34.6 | 2,3,5,6 |
| 35 | **51** | 75 | G2 | > 50% | yes | IA | postmenopausal | 30.4 | 1,6 |
| 36 | **52** | 75 | G2 | < 50% | yes | IA | postmenopausal | 48.9 | 1,2,3,4,5,6 |
| 37 | **53** | 50 | G3 | < 50% | yes | IA |  |  | 1,2,3,4,5,6 |
| 38 | **54** | 71 | G1 | < 50% | no | IA | postmenopausal | 41.1 | 1,2,3,5,6 |
| 39 | **55** | 75 | serous | > 50% | yes | IIIC1 | postmenopausal | 24.0 | 3,5,6 |
| 40 | **56** | 55 | G1 | < 50% | no | IA | postmenopausal | 38.1 | 1,2,3,4,5,6 |
| 41 | **57** | 43 | G1 | < 50% | no | IA | premenopausal | 44.6 | 1,2,3,6 |
| 42 | **58** | 68 | G2 | < 50% | no | IA | postmenopausal | 34.9 | 2,5,6 |
| 43 | **59** | 48 | G1 |  | yes | IVB | premenopausal | 20.5 | 3,4,5 |
| 44 | **60** | 56 | G1 | < 50% | no |  | postmenopausal | 32.7 | 2 |
| 45 | **61** | 83 | G1 | < 50% | no | IA | postmenopausal | 33.1 | 2,3,4,5 |
| 46 | **62** | 59 | G1 | < 50% | no | IA | postmenopausal | 37.5 | 2,3,4,5 |
| 47 | **63** | 66 | G1 | < 50% | no | IA | postmenopausal | 34.6 | 2,3,4,5 |
| 48 | ***64*** | 66 | G1 | < 50% | no | IA | postmenopausal | 25.5 | 2,3,5 |
| 49 | **65** | 80 | carcinosarcoma | > 50% | yes | IB | postmenopausal | 28,1 | 2,3,5 |
| 50 | **66** | 72 | G1 | < 50% | no | IA | postmenopausal | 27,5 | 2,3,5 |
| 51 | **67** | 44 | G1 | < 50% | no | IA | premenopausal | 29.0 | 2 |
| 52 | **68** | 45 | G1 | < 50% | no | II | premenopausal | 20.8 | 2,3 |
| 53 | **69** | 72 | G1 | < 50% | no | IA | postmenopausal | 29.4 | 2 |
| 54 | **70** | 55 | G3 | > 50% |  | IB | postmenopausal | 35.6 | 2,3 |
| 55 | **71** | 48 | serous | < 50% | no | IA | premenopausal |  | 2,3 |

G1 - G3 endometrioid adenocarcinoma

**Supplementary Table 2. Details of the investigated and reference genes.**

| **Gene Symbol** | **Assay ID** | **Gene Name** |
| --- | --- | --- |
| *STS* | Hs00165853_m1 | steroid sulfatase (microsomal), isozyme S |
| *CYP19A1* | Hs00240671_m1 | cytochrome P450, family 19, subfamily A, polypeptide 1 |
| *HSD17B1* | Hs00166219_g1 | hydroxysteroid (17-beta) dehydrogenase 1 |
| *HSD17B14* | Hs00212233_m1 | hydroxysteroid (17-beta) dehydrogenase 14 |
| *PPIA* | Hs99999904_m1 | peptidylprolyl isomerase A (cyclophilin A) |
| *HPRT1* | Hs99999909_m1 | hypoxanthine phosphoribosyltransferase 1 (Lesch-Nyhan syndrome) |
| *POLR2A* | Hs00172187_m1 | polymerase (RNA) II (DNA directed) polypeptide A, 220kDa |

**Supplementary Table 3. Description of antibodies and protocols for Western blotting.**

| **Information on Antibodies** | | | |
| --- | --- | --- | --- |
| **Antigen, type of antibodies** | **Manufacturer,**  **Catalogue #,**  **Batch #,**  **name of the source** | **Dilution used** | **Secondary antibody** |
| STS,  rabbit,  polyclonal | Provided by Dr. Schuller (Dibbelt and Kuss 1986, Dibbelt *et al.* 1989) | 1:5 000 in TTBS with 5% BSA;  2 h at 4 °C | Peroxidase-conjugated goat anti-rabbit IgG + IgM [H + L], 1:5 000 (Jackson ImmunoResearch Laboratories Inc., USA, 111-035-045) in TTBS with 2.5% BSA; 2 h at 4 °C. |
| HSD17B2,  rabbit,  polyclonal | Provided by Dr. Husen, Solvay Pharmaceuticals, Hannover, Germany  (Dassen et al. 2007) | 1:1 250 in TTBS with 5% non-fat milk powder; overnight at 4 °C | Peroxidase-conjugated goat anti-rabbit IgG + IgM [H + L], 1:10 000 (Jackson ImmunoResearch Laboratories Inc., USA, 111-035-045); in TTBS with 5% non-fat milk powder; 1 h at 4 °C. |
| SULT1E1,  rabbit, polyclonal | Sigma Aldrich Germany, Cat. # HPA028728,  Lot R28328 | 1:250 in TTBS with 5% non-fat milk powder; overnight at 4 °C | Peroxidase-conjugated goat anti-rabbit IgG + IgM [H + L], 1:5 000 (Jackson ImmunoResearch Laboratories Inc., USA, 111-035-045) ) in TTBS with 1% non-fat milk powder; 1 h at 4 °C. |
| HSD17B1,  rabbit monoclonal | Abcam; Cambridge, UK, Cat. # EP1682Y, ab51045, Lot GR120943-6 | 1:1 000 in TTBS with 1% nonfat milk powder; overnight at 4 °C | Peroxidase-conjugated goat anti-rabbit IgG + IgM [H + L], 1:10 000 (Jackson ImmunoResearch Laboratories Inc., USA, 111-035-045) in TTBS with 1% non-fat milk powder; 2 h at 4 °C. |
| HSD17B1,  rabbit polyclonal | Provided by Dr. Husen, Solvay Pharmaceuticals, Hannover, Germany  (Dassen et al. 2007) | 1:15 000 in TTBS with 2% nonfat milk powder; overnight at 4 °C | Peroxidase-conjugated goat anti-rabbit IgG + IgM [H + L], 1:10 000 (Jackson ImmunoResearch Laboratories Inc., USA, 111-035-045) in TTBS with 5% non-fat milk powder; 2 h at 4 °C. |
| GAPDH,  mouse, polyclonal | Sigma Aldrich, Germany Cat. # G8795, Lot 086K4832 | 1:2 500 in TTBS with 1% non-fat milk powder  overnight at 4 °C | Peroxidase-conjugated goat anti-mouse IgG [H + L], 1:5 000 (Jackson ImmunoResearch Laboratories Inc., USA, 115-035-062) in TTBS with 1% non-fat milk powder; 2 h at 4 °C. |

**Supplementary Table 4. The immunohistochemical staining scores.**

|  | **Score (Intensity x Percentage)** | | | | | | **Score (Intensity)** | |
| --- | --- | --- | --- | --- | --- | --- | --- | --- |
|  | **STS** | | **HSD17B2** | | **SULT1E1** | | **HSD17B1** | |
| Sample | Control | Tumor | Control | Tumor | Control | Tumor | Control | Tumor |
| 1 | 3 | 15 | 0 | 30 | 30 | 0 | 0.5 | 0.5 |
| 3 | 90 | 90 | 0 | 100 |  |  | 0.5 | 0.5 |
| 4 | 80 | 90 | 0 | 200 |  |  | 0.5 | 0.5 |
| 6 | 55 | 45 | 25 | 200 | 30 | 0 | 0.5 | 0 |
| 9 | 95 | 90 | 50 | 100 |  |  | 0.5 | 1 |
| 10 | 90 | 90 | 50 | 150 |  |  | 0.5 | 0.5 |
| 11 | 90 | 135 | 0 | 200 |  |  | 0 | 0.5 |
| 13 |  |  |  |  |  |  | 0.5 | 0.5 |
| 19 | 90 | 40 |  |  | 0 | 0 | 0.5 | 0.5 |
| 21 | 60 | 5 | 100 | 100 | 70 | 0 | 0 | 0 |
| 22 | 90 | 90 | 0 | 200 |  |  | 0.5 | 1 |
| 23 | 190 | 135 | 150 | 300 |  |  | 0.5 | 0,5 |
| 24 | 10 | 0 | 50 | 100 | 0 | 0 | 0 | 1 |
| 25 | 135 | 90 | 50 | 300 |  |  | 0 | 0.5 |
| 26 | 90 | 90 |  |  |  |  | 0.5 | 0.5 |
| 30 | 90 | 90 |  |  |  |  | 0.5 | 0.5 |
| 31 | 180 | 90 |  |  |  |  | 0 | 0.5 |
| 34 | 5 | 3 | 100 | 200 | 0 | 0 | 0.5 | 0.5 |
| 35 | 0 | 5 | 50 | 90 | 0 | 0 |  |  |
| 38 | 0 | 30 | 100 | 80 | 0 | 0 | 1 | 0 |
| 40 | 10 | 5 | 50 | 80 | 0 | 0 | 1 | 0.5 |
| 42 | 13 | 15 | 50 | 80 | 0 | 0 | 0.5 | 0.5 |
| 44 | 70 | 120 | 50 | 100 | 30 | 135 | 0.5 | 1 |
| 46 | 60 | 5 | 50 | 100 | 70 | 0 | 0.5 | 0.5 |
| 47 | 6 | 25 | 150 | 200 | 40 | 0 | 1 | 0.5 |
| 49 | 80 | 30 | 100 | 150 | 40 | 30 | 0.5 | 0 |
| 50 | 20 | 30 | 100 | 43 | 0 | 50 | 0.5 | 0.5 |
| 52 | 80 | 135 | 100 | 200 | 0 | 80 | 0.5 | 0.5 |
| 53 | 135 | 5 | 100 | 20 | 90 | 70 | 0.5 | 0.5 |
| 54 | 135 | 50 | 100 | 100 | 90 | 105 | 0.5 | 0.5 |
| 56 | 135 | 10 | 50 | 100 | 90 | 0 | 1 | 0 |
| 57 | 3 | 105 | 150 | 100 | 35 | 52.5 | 0.5 | 0.5 |
| 58 | 80 | 40 | 150 | 150 | 90 | 0 | 0.5 | 0.5 |
| 60 | 45 | 60 | 75 | 100 | 40 | 92.5 | 0 | 0.5 |
| 61 | 60 | 90 | 100 | 60 |  |  |  |  |
| 62 | 90 | 103 | 150 | 200 | 102.5 | 135 | 0.5 | 1 |
| 63 | 90 | 30 | 100 | 0 |  |  | 0.5 | 0 |
| 64 | 80 | 15 | 100 | 200 | 80 | 20 | 0.5 | 0.5 |
| 65 | 60 | 0 | 100 | 10 | 70 | 0 | 0.5 | 0.5 |
| 66 | 35 | 3 | 150 | 60 | 20 | 0 | 0 | 0.5 |
| 67 | 135 | 25 | 150 | 150 | 135 | 10 | 0 | 0.5 |
| 68 | 40 | 15 | 150 | 60 | 70 | 0 |  |  |
| 69 | 65 | 20 | 100 | 50 | 80 | 0 | 0 | 0.5 |
| 70 | 40 | 40 | 150 | 100 | 30 | 0 | 0 | 0.5 |
| 71 | 40 | 30 | 150 | 160 | 0 | 0 | 0 | 1 |
