# Supplementary material for: The Significance of the Sulfatase Pathway for Local Estrogen Formation in Endometrial Cancer
Source: Front Pharmacol. 2017 Jun 23;8:368. doi: 10.3389/fphar.2017.00368 (PMC5481366; doi:10.3389/fphar.2017.00368)
Supplement: Supplementary file 2 [file DataSheet1.PDF]

Supplementary Figure 1

A-dione metabolism in EC tissue samples

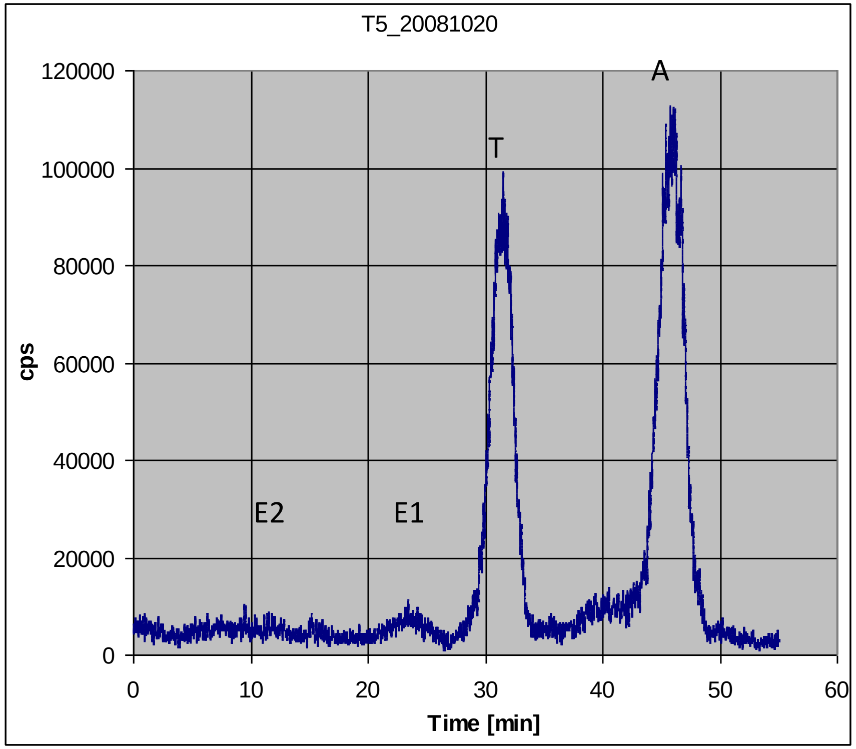

Sample	Testosterone fmol/mg prot.	Estrone (E1) fmol/mg prot.
T0	3.33	0.09
T1	0.39	0.08
T5	1.66	0.07
T6	1.61	0.50
T11	5.94	0.16
T13	2.04	0.71
T14	0.19	0.08
T16	1.34	0.05
T18	3.71	0.11
median	1.66	0.09

Metabolism of 10 nM ^{3}H A-dione was studied in nine EC tissue samples (≈ 30 mg homogenized tissue) in PBS buffer, pH 7.4,with addition of 5 mM NADPH. The incubation was performed for 22 h at 37°C. The products were separated by HPLC. The representative chromatogram for sample 5 is shown, together with a table with data on Testosterone and Estrone levels in fmol/mg protein.
